# Fitness Burden for the Stepwise Acquisition of First- and Second-Line Antimicrobial Reduced-Susceptibility in High-Risk ESKAPE MRSA Superbugs

**DOI:** 10.3390/antibiotics14030244

**Published:** 2025-02-28

**Authors:** Eleonora Chines, Gaia Vertillo Aluisio, Maria Santagati, Maria Lina Mezzatesta, Viviana Cafiso

**Affiliations:** 1 Department of Biomedical and Biotechnological Sciences, University of Catania, 95123 Catania, Italy; eleonora.chines01@universitadipavia.it (E.C.); gaia.vertilloaluisio@unict.it (G.V.A.); m.santagati@unict.it (M.S.); mezzate@unict.it (M.L.M.); 2 PhD National Program in One Health Approaches to Infectious Diseases and Life Science Research, Department of Public Health, Experimental, and Forensic Medicine, University of Pavia, 27100 Pavia, Italy

**Keywords:** fitness costs, DAP-R, hGISA, GISA, genomics, virulence, growth, competitiveness, *Galleria mellonella* killing

## Abstract

**Background:** The fitness costs (FCs) of antimicrobial resistance (AMR) are crucial issues in antimicrobial resistance (AMR) onset, spread, and, consequently, public health. In *Staphylococcus aureus*, AMR can induce significant FCs due to slow growth, low competitiveness, and virulence. Here, we investigated the genomics and FCs emerging for progressively acquiring daptomycin (DAP) and glycopeptide (GLY) reduced susceptibility in MRSA. **Methods:** Genomics was carried out using Illumina-MiSeq Whole-genome sequencing and bioinformatics. The biological FCs of isogenic MRSA strain pairs progressively acquiring DAP and GLY-reduced susceptibility, under DAP/GLY mono or combined therapy, were performed by in-vitro independent and competitive mixed growth, phenotypic in-vitro virulence analysis, and in-vivo *G. mellonella* larvae killing. **Results:** Genomics evidenced four different extremely resistant high-risk clones, i.e., ST-5 N315 HA-MRSA, ST-398 LA-MRSA, ST-22 USA-100 HA-EMRSA-15, and ST-1 MW2 CA-MRSA. In-vitro fitness assays revealed slow growth, lower competitiveness, and reduced virulence, predominantly in *Galleria mellonella* killing ability, in DAP-S hGISA, DAP-R GSSA, DAP-R hGISA, and DAP-R GISA strains. **Conclusions:** The occurrence of glycopeptide and daptomycin reduced susceptibility conferred increasing FCs, paid as a gradual reduction in virulence, competitiveness, and slow growth performance.

## 1. Introduction

Fitness is defined as the ability to grow prosperously in an environment characterized by nutrient availability, physicochemical conditions, and the presence of antimicrobials, as proposed by Botelho et al. (2019) [1]. It is closely dependent on growth rate, inter-bacterial competitiveness and is interconnected with virulence. This interplay represents a crucial point in the behavior and impact of pathogens [2]. The chance of survival and spread increases with the ability to infect a host. A slower growth in the host could determine lower/decreased virulence and vice versa. Notably, this observation likely matches what happens in acute infections [3,4,5] and, more so, in chronic ones in which slow growth and reduced virulence seem to be the hallmark of a successful adaptation [6,7,8].

The development of multi-drug resistance in the ESKAPE (*Enterococcus faecium*, *Staphylococcus aureus*, *Klebsiella pneumoniae*, *Acinetobacter baumannii*, *Pseudomonas aeruginosa*, and *Enterobacter* spp.), such as the WHO high-priority pathogen Methicillin-Resistant *Staphylococcus aureus* (MRSA) [9], often occurs with fitness costs (FCs) that can negatively affect the bacteria’s growth rate, inter-bacterial competitive ability, and virulence compared to susceptible counterparts [10]. The level of these costs significantly influences the rate at which resistance spreads, the stability, and how rapidly resistance can decline if antibiotic use is reduced [11,12,13]. Their relationship is complex and context-dependent.

Highly virulent bacteria well-adapted to the host are well-fit to cause infection; however, they might have a reduced transmission rate as they kill their host too rapidly [2,11]. *S. aureus* produces a great pool of virulence factors, i.e., toxins, pigments, proteases, cytotoxicity, and biofilm formation, coherently with its ability to cause different infections [14,15]. Consequently, together with growth and competitiveness, a complex interplay of numerous virulence-related factors can contribute to fitness.

The acquisition of antimicrobial resistance (AMR) is a gain-in function frequently associated with gain-off counterparts as biological fitness costs [16]. These costs can result from mutations (mutation-driven resistance), the acquisition of a gene (or set of genes) conferring a new function, the acquisition of mobile genetic elements (MGEs) such as plasmid-carrying resistance genes (horizontal-acquired resistance) [17], and metabolic shifts towards adaptive resistance pathways (adaptive pathway-acquired resistance) [18].

Glycopeptides (GLY)—vancomycin (VAN), teicoplanin (TEC), telavancin (TEL), and dalbavancin (DAL)—are the first-line treatment for severe MRSA infection, while daptomycin (DAP), linezolid, and tigecycline are among the second-line or last-resort therapeutic options [19]. Unfortunately, MRSA can develop reduced susceptibility (RS) to glycopeptides, still at a low rate [20,21], leading to the emergence of glycopeptide-intermediate-resistant *S. aureus* (GISA) with a vancomycin MIC of 4–8 mg/L, or heterogeneous GISA (hGISA) with a vancomycin MIC of ≤2 mg/L, often associated with DAP-RS with a MIC > 1 mg/L.

We aimed to investigate the genomics and FCs in six clinical and reference isogenic MRSA, members of predominant high-risk clones, progressively acquiring DAP and GLY-RS, under DAP/GLY mono or combined therapy, i.e., one DAP-susceptible (DAP-S) heterogeneous glycopeptide-intermediate-resistant *S. aureus* (hGISA) vs. its DAP-S glycopeptide-susceptible *S. aureus* (GSSA) strain pair, one DAP-R (DAP-reduced susceptible) GSSA vs. DAP-S GSSA strain pair, two DAP-R hGISA vs. DAP-S GSSA strain pairs, and two DAP-R glycopeptide-intermediate-resistant *S. aureus* (GISA) vs. DAP-S GSSA strain pairs.

## 2. Results

The genomic characterization and FCs generated in MRSA, progressively acquiring exclusive hGISA, DAP-RS, or GLY and DAP cross-resistance at different rates, were evaluated versus their isogenic susceptible parents (intra-strain pairs) and among the different strain pairs (inter-strain pairs).

This double-check investigation confers great strength to the data, representing a significant and unique viewpoint to evaluate FCs without bias due to different genomic backgrounds despite their different predominant spreading MRSA lineages.

In detail, in the comparison of the isogenic R vs. S strains within the same strain pair, we looked for FCs acquired with the occurrence of specific resistance in the same genomic background. In contrast, in the comparison among the different strain pairs, we investigated FCs due to the progressive acquisition of different DAP- and GLY-reduced susceptibility rates in different genomic lineages.

### 2.1. Epidemiological and Genomic Typing, Resistome, and Virulome

The genomic characterization showed four different extremely resistant high-risk clones. The clinical reference (ref) DAP-S hGISA and DAP-S GSSA 1-R/S were ST-5 N315 hospital-acquired MRSA (HA-MRSA), spatype-2, agr-II, and SCCmecIIa; the clinical DAP-R GSSA and DAP-S GSSA 2-R/S were ST-5 N315 HA-MRSA, spatype-t2, agr-II, and SCCmecII; the clinical DAP-R hGISA/DAP-S GSSA 3-R/S were ST-398 livestock-associated MRSA (LA-MRSA), spatype-t1939, agr-I, and SCCmecIVa; the clinical DAP-R hGISA and DAP-S GSSA 4-R/S were ST-22 USA-100 HA-EMRSA-15, spatype-t25, agr-I, and SCCmecIV; the clinical DAP-R GISA and DAP-S GSSA 5-R/S were ST-1 MW2 community-acquired MRSA (CA-MRSA), USA-400, spatype-t127, agr-III, and SCCmecIVa; the clinical reference DAP-R GISA and DAP-S GSSA 6-R/S were ST-5 N315 HA-MRSA, spatype-2, agr-II, and SCCmecIIa (Table S1). Resistomes, virulomes, genomic islands, MGEs (plasmids, transposons, insertion sequences, pathogenicity islands), and resistome/AMR-related non-synonymous SNPs (nsSNPs) were reported in Table S2.

In detail, the 1-R mobilome included the rep22 plasmid, ISSau4, and ISLgar5 (this last one was acquired vs. 1-S) insertion sequences, as well as the *Tn*554 transposon. Genomics revealed 11 horizontal-acquired virulence-resistance genomic islands (GIs). Resistomics found *mecA* (Beta-lactams),* bleO* (Glycopeptides), *erm(A)* (Macrolide/Lincosamide), and *tet(M)* (Tetracycline) resistance-associated genes, as well as S80F GrlA and S84L GyrA (Ciprofloxacin) (acquired vs. 1-S) resistance-related AA-changes.

The 2-R mobilome was constituted by rep20 and rep22 plasmids, ISSau4 insertion sequences, and the *Tn*554 transposon. Genomics evidenced three horizontal-acquired virulence-resistance GIs, as well as a resistome including *mecA* and *blaZ* (Beta-lactams),* bleO* (Glycopeptides), *erm(A)* (Macrolide/Lincosamide), *aadD*, and *ant(9)-Ia* (Aminoglycoside) resistance-associated genes, together with S80F *GrlA* and S84L GyrA (Ciprofloxacin) and A477D RpoB (Rifampicin) resistance-related AA changes.

The 3-R mobilome carried rep10, repUS43, and rep15 plasmids; an ISSau8 insertion sequence; and the *Tn*6009 transposon. Genomics highlighted nine horizontal-acquired virulence-resistance GIs. The resistome included *mecA* (Beta-lactams),* erm(C)-vga(A)V-fexB* (Macrolide/Lincosamide),* tet(M)* (Tetracycline), *aac(6*′*)-aph(2*″*)* (Aminoglycoside), and *fosD* (Fosfomycin) resistance-associated genes, together with S80F GrlA (Ciprofloxacin) and A477D RpoB (Rifampicin) resistance-related AA changes.

The 4-R mobilome evidenced rep5, rep10, and rep20 plasmids and IISSau2-ISSau5-ISSau6 insertion sequences. The genome included seven horizontal-acquired virulence-resistant GIs. Resistomics showed *mecA-bla*Z (Beta-lactams) and *erm(C)* (Macrolide/Lincosamide) resistance-associated genes, together with S80F GrlA and S84L GyrA (Ciprofloxacin) resistance-related AA changes.

The 5-R mobilome had rep7a, rep7c rep5a, rep16, and rep10 plasmids. The genome presented six horizontal-acquired virulence-resistant GIs. The resistome included *mecA* (Beta-lactams),* erm(C)* (Macrolide/Lincosamide), and *tet(K)* (Tetracycline) resistance-associated genes, associated with S80F GrlA and S84L GyrA (Ciprofloxacin) resistance-related AA changes.

The 6-R mobilome included the rep22 plasmid, ISSau4 and ISLgar5 (this last one was acquired vs. 1-S) insertion sequences, and the *Tn*554 transposon. The genome contained 11 horizontal-acquired virulence-resistance GIs and a resistome comprising *mecA* (Beta-lactams),* bleO* (Glycopeptides), *erm(A)* (Macrolide/Lincosamide), and *tet(M)* (Tetracycline) resistance-associated genes, associated with S80F GrlA and S84L GyrA (Ciprofloxacin) (acquired vs. 1-S) resistance-related AA-changes.

Of note, our data evidenced a small virulome in the clinical DAP-R hGISA 3-R/S (48 virulence genes; Virulome_score: +1/3) and 4-R/S (56 virulence genes; Virulome_score: +1/3), a medium virulome in the clinical DAP-R GISA 5-R/S (64 virulence genes; Virulome_score: +2/3), and a large virulome in all remaining strain pairs (72, 75, and 77 virulence genes, respectively; Virulome_score: +3/3), as shown in Table S3.

### 2.2. Fitness Costs

The FCs were evaluated as the changes in growth performance in independent-growth, inter-bacterial competitiveness in mixed-growth, and virulence as virulome size, in-vitro and in-vivo virulence, comparing the R vs. S strains.

#### 2.2.1. Growth Performance

Growth performances were evaluated by growth curves, generation times (gT), and growth rates (r) in independent growth (Table S4). A growth score burden (G_score), in relation to the rate of growth-performance changes [high (3), medium (2), low (1)] was assigned.

Comparing intra-strain pair, independent growth revealed statistically significant changes in growth performance in all R strains vs. their susceptible parents.

**Figure 1:**
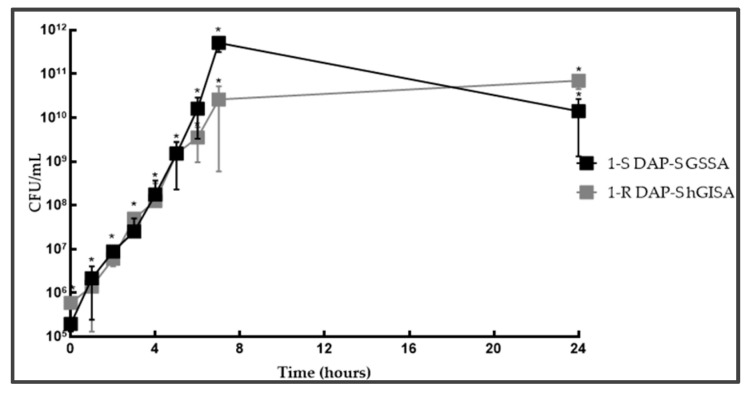
1-S/R independent growth curve. Legend: * statistically significant (*p* < 0.05).

In detail, in DAP-S hGISA 1-R vs. 1-S, a 1 h longer lag-phase was evidenced (Table S4, Figure 1). Consequentially, the exponential phase started 1 h later. In addition, it had a slow-growing exponential trend characterized by a growth rate that doubled in 2 h rather than 1 h, as found in the susceptible counterpart. The late stationary phase did not show a real plateau phase (present in 1-S) but rather a slower growth with a long gT (11 h:29 min) and low r (0.06) (G_score: +1/3).

**Figure 2:**
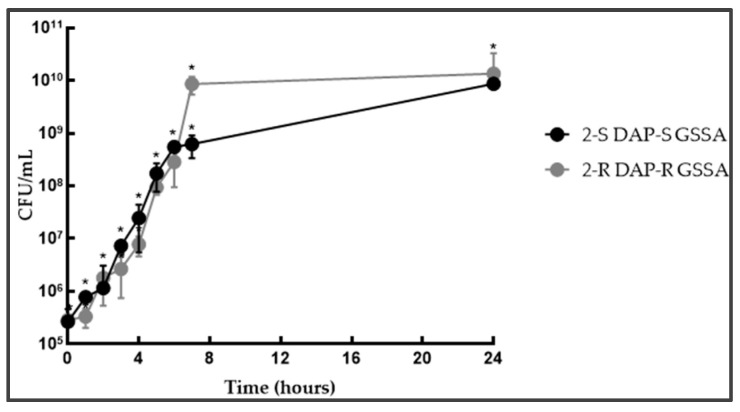
2-S/R independent growth curve. Legend: * statistically significant (*p* < 0.05).

In DAP-R GSSA 2-R vs. 2-S, the lag-phase was 2 h longer (Table S4, Figure 2). The exponential phase started later; however, no changes in the exponential hourly gT trend and stationary phase were observed (G_score: +2/3).

**Figure 3:**
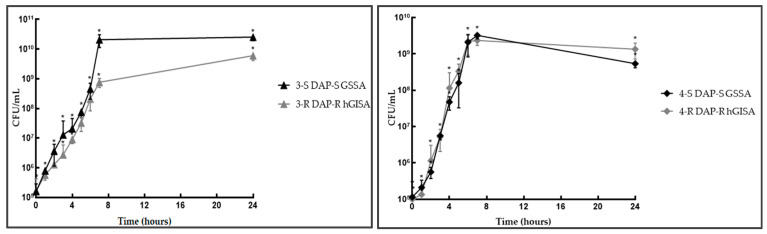
3-S/R and 4-S/R independent growth curves. Legend: * statistically significant (*p* < 0.05).

In DAP-R hGISA 3/4-R vs. 3/4-S, the lag-phase shifted from 1 to 3 growth-hours in 3-R and from 2 to 4 growth-hours in 4-R, thus extending it by 2 h in both strain pairs (Table S4, Figure 3). The exponential phase was characterized by longer hourly gTs (about 30 min instead of about 20 min), leading to a unique doubling-growth step at 6 growth-hours in 3R and a short exponential phase (2 h) in 4-R. The stationary phase was wide-ranging in different strains, i.e., no changes were recovered in 3-R, whilst an early entry (at 7 h) and a reduced plateau phase (gT: −17 h, r: 0.04) were found in 4-R (G_score: +2/3).

**Figure 4:**
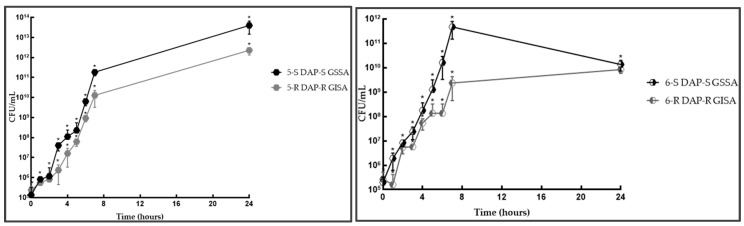
5-S/R and 6-S/R independent growth curves. Legend: * statistically significant (*p* < 0.05).

In DAP-R GISA (5/6-R) vs. 5/6-S, the lag-phase was extended from 2 to 5 growth-hours in 5-R and 3 to 6 growth-hours in 6-R (Table S4, Figure 4). This indicates that the lag-phase duration was 3 h longer in both strain pairs. The exponential phase was significantly delayed at 6 h in 5-R and 7 growth-hours in 6-R, even though no shifts in the exponential hourly gT were detected. The stationary phase maintained a similar trend to its susceptible counterpart in 5-R, whereas it showed a reduced plateau in 6-R (gT: 9 h:55 min, r: 0.069) (G_score: +3/3).

Comparing clinical independent-growth inter-strain pairs, the growth parameters showed different and/or increasing growth alteration that was progressively acquired in DAP-R GSSA (G_score: +1/3) < DAP-S hGISA (G_score: +2/3) = DAP-R hGISA (G_score: +2/3) < DAP-R GISA (G_score: +3/3).

#### 2.2.2. Competitiveness

Competitiveness was evaluated by a statistically significant reduction in the population density of the R strains vs. S parents in competitive mixed growth, evidenced by an S strain out-competing. A competitiveness score (C_score), based on the rate of competitiveness changes [huge (5), high (4), medium (3), low (2), very low (1)] was assigned.

Comparing the intra-strain pairs, clinical DAP-S hGISA 1-R, DAP-R GSSA 2-R, DAP-R hGISA 3/4-R, and DAP-R GISA 5/6-R strains evidenced an increasing reduction in competitiveness versus their susceptible counterparts in mixed-growth, as shown in Table S5.

The DAP-R GSSA 2-R maintained the highest competitiveness. In 2-R/S co-cultures, a transitory and very low DAP-S GSSA 2-S outcompeting was evidenced vs. DAP-R GSSA 2-R, with only 2S 1 LogCFU/mL increasing in population density at 2–5 h of growth (C_score: +1/5).

In contrast, independently of the DAP-R acquisition, hGISA (1-R, 3-R, and 4-R) showed a high/medium rate of competitiveness. A stable long-term and low outcompeting, prevalently with a 1 or occasionally 2 LogCFU/mL growth increase between 3 and 24 growth-hours, was recorded in clinical DAP-S GSSA (3-S,4-S) vs. DAP-R hGISA (3-R,4-R) (C_score: +2/5).

A stable long-term, and moderately reduced competitiveness, mainly a 2 or occasionally 1 LogCFU/mL reduction in population density from 2 to 24 h of growth, was found in Ref DAP-S hGISA (1-R) vs. DAP-S GSSA (1-S) (C_score: +3/5).

Finally, both DAP-R GISA (5-R/6-R) evidenced a stable long-term and large-scale reduced competitiveness leading to the lowest competitiveness, with a poor population density with a decrease ranging from 1 to 4 LogCFU/mL during 2–24 h of growth vs. susceptible counterparts. Clinical and Ref DAP-S GSSA strains (5-S/6-S) significantly outcompeted their DAP-R GISA strains (5R/6R) (C_score: +4–5/5).

Comparing the inter-strain pairs, mixed-growth parameters revealed that, in the absence of antimicrobials, the lowest competitiveness was in both DAP-R GISA strain pairs (C_score: +4–5/5) and DAP-S hGISA (C_score: +3/5), in DAP-R hGISA (C_score: +2/5) with a medium strain/lineage-dependent competitiveness, and in DAP-R GSSA (C_score: +1/5) maintaining the best competitiveness.

#### 2.2.3. Virulence

Virulence FCs were determined as a statistically significant reduction in virulence in R strains vs. S parents. The phenotypic in-vitro production of a set of relevant virulence factors, including biofilm formation, hemolysin activity, staphyloxanthin production, protease activity, and colony spreading along with the in-vivo killing ability in *Galleria mellonella* larvae was evaluated. in-vitro and in-vivo assays were investigated to include all possible mechanisms of virulence detectable in in-vitro and in-vivo models.

An in-vitro virulence score (in-vitro V_score) was assigned based on the sum of the scores of in-vitro virulence factor production [high strong (+4), strong (+3), medium (+2), weak (+1) or negative (0)].

Similarly, a killing score (K_score) was assigned to the in-vivo killing ability rate (+7/+1).

The in-vitro V_reduction_score and in-vivo V_reduction_score were determined by subtracting the V_score of the S strain from the R one.

A virulence score (V_score) was assigned based on the virulome size and the sum of the rates of in-vitro and in-vivo virulence changes [huge (5), high (4), medium (3), low (2), very low (1), null (0)].

##### In-Vitro Virulence Factor Production

Comparing intra-strain pair DAP-S hGISA 1-R vs. 1-S, a phenotypic virulence analysis revealed that DAP-S hGISA 1-R decreased to weaken the production of biofilm, staphyloxanthin, and colony spreading (in-vitro V_score: +3/10) vs. the strong biofilm, medium staphyloxanthin, and colony spreading of DAP-S GSSA 1-S production (in vitro V_score: +7/10). In-vitro V_reduction_score: +4/7.

Analyzing intra-strain pair DAP-R GSSA 2-R vs. 2-S, DAP-R GSSA 2-R decreased to a weak staphyloxanthin production and to a medium colony-spreading ability (in-vitro V_score: +5/10) vs. the strong staphyloxanthin and colony-spreading producer, weak biofilm, and alpha-hemolysin production of DAP-S GSSA 2-S (in-vitro V_score: +8/10; Table 1 and Table S6, Figures S1–S3). In-vitro V_reduction_score: +3/7.

Evaluating intra-strain pair DAP-R hGISA 3-R vs. 3-S, DAP-R hGISA 3-R was very similar to its susceptible counterpart, having only a weaker colony-spreading (in-vitro V_score: +4/10) vs. medium colony spreading, weak alpha-hemolytic, and weak protease in DAP-S GSSA 3-S (in-vitro V_score: +4/10; Table 1 and Table S6, Figures S1–S3). In-vitro V_reduction_score: 0/7.

Assessing intra-strain pairDAP-R hGISA 4-R vs. 4-S, DAP-R hGISA 4-R lacked protease production and decreased to weaken the colony-spreading ability (in-vitro V_score: +3/10) vs. medium colony spreading and a weak alpha-hemolytic, staphyloxanthin, and protease producer DAP-S GSSA 4-S (in-vitro V_score: +5/10; Table 1 and Table S6, Figures S1–S3). In-vitro V_reduction_score: +2/7.

Relating intra-strain pair DAP-R GISA 5-R vs. 5-S, DAP-R GISA 5-R decreased colony spreading and staphyloxanthin activity to weaken production and also had a lack of alpha- and delta-hemolysis and protease production (in-vitro V_score: +2/10) vs. the highly strong colony spreading, medium staphyloxanthin formation, weak alpha-hemolytic, delta-hemolytic and protease producer (in-vitro V_score: +9/10) in DAP-S GSSA 5-S (Table 1 and Table S6, Figures S1–S3). In-vitro V_reduction_score: +7/7.

Comparing intra-strain pair DAP-R GISA 6-R vs. 6-S, DAP-R GISA 6-R strains decreased to weaken the production of biofilm, staphyloxanthin, and colony spreading (in-vitro V_score: +3/10) vs. the strong biofilm, medium staphyloxanthin, and colony-spreading producer DAP-S GSSA 6-S (in-vitro V_score: +7/10; Table 1 and Table S6, Figures S1–S3). In-vitro V_reduction_score: +4/7.

Comparing inter-strain pairs, in-vitro virulence assays revealed that the highest reduction in virulence was in both DAP-R GISA 5-R (in-vitro V_reduction_score: +7/7) > DAP-R GISA 6-R = DAP-S hGISA 1-R (in-vitro V_reduction_score: +4/7) > DAP-R GSSA 2-R (in-vitro V_reduction_score: +3/7) > DAP-R hGISA 4-R (in-vitro V_reduction_score: +2/7) and DAP-R hGISA 3-R (in-vitro V_reduction_score: 0/7).

In the USA300 and MW2 control strains, the virulence factor production showed an in-vitro V_score: +7 and an in-vitro V_score: +10, respectively, in agreement with the virulence production levels shown in Table 1 and Table S6 and Figures S1–S3.

##### In-Vivo Killing in *Galleria mellonella* Larvae

The killing assays in *Galleria mellonella* larvae showed different rates of killing ability in the different strain pairs, as follows.

DAP-S hGISA 1-R had an in-vivo poorly reduced virulence, maintaining its intrinsic high virulence. DAP-S hGISA 1-R slightly decreased the fast and full (all larvae died) killing ability at 48 h, instead of 24 h, as seen in DAP-S GSSA 1-S. Killing_score (K_score): +6K in 1-R vs. +7K in 1-S. In-vivo V_reduction_score: +1/5.

The DAP-R GSSA strain 2-R maintained a stable and high in-vivo virulence. DAP-R GSSA 2R conserved the fast and full killing ability of all larvae, within 48 h, as seen in DAP-S GSSA. K_score: +6K in 2-R vs. +6K in 2-S. In-vivo V_reduction_score: 0/5.

DAP-R hGISA 3-R had a stable and modest in-vivo virulence. DAP-R hGISA 3-R (as DAP-S GSSA 3-S) evidenced a very slow and partial ability to kill larvae, the 20% of surviving larvae were still detected after more than 120 h. K_score: +3K in 3-R vs. +3K in 3-S. In-vivo V_reduction_score: 0/5.

DAP-R hGISA 4-R had drastically reduced in-vivo virulence, acquiring the lowest in-vivo virulence. DAP-R hGISA 4-R had a slower and partial (60%) killing ability only after more than 120 h vs. the fast and full killing ability, within 48 h, in DAP-S GSSA 4-S. K_score: +1K in 4-R vs. +6K in 4-S. In-vivo V_reduction_score: +5/5.

DAP-R GISA 5-R had a high/medium-reduced virulence, achieving a low in-vivo virulence. A slower (>120 h) and partial (40%) killing ability was recorded in 5-R vs. the intermediate and full killing ability, within 72 h, in 5-S. In-vivo K_score: +2K in 5-R vs. +5K in 5-S. In-vivo V_reduction_score: +3/5.

DAP-R GISA 6-R had a poorly reduced in vivo virulence, maintaining discreet virulence. DAP-R GISA 6-R decreased the full killing ability, within 48 h vs. 24 h, in the DAP-R GSSA 6-S strain. Killing_score (K_score): +6K in 6-R vs. +7K in 6-S. n-vivo V_reduction_score: +1/5.

Similar behavior was shown in the MW2 control strain with a fast and full killing ability within 48 h (K_score: +6). The USA300 control strain had a slow and partial ability to kill larvae (K_score: +4), and after more than 72 h, surviving larvae (20%) were still recovered.

Comparing inter-strain pairs, in-vivo virulence assays revealed that the highest virulence reduction was found in DAP-R hGISA 4-R (in-vivo V_reduction_score: +5/5) > DAP-R GISA 5-R (in-vivo V_reduction_score: +3/5) > DAP-R GISA 6-R (in-vivo V_reduction_score: +1/5) = DAP-S hGISA 1-R (in-vivo V_reduction_score: +1/5) > DAP-R hGISA 3-R=DAP-R GSSA and the 2-R strains (in-vivo V_reduction_score: 0/5; Table 2).

The survival percentage of *G. mellonella* larvae of the untreated, sham injection, and control groups was 100%.

### 2.3. Fitness Cost Burden

**Figure 5:**
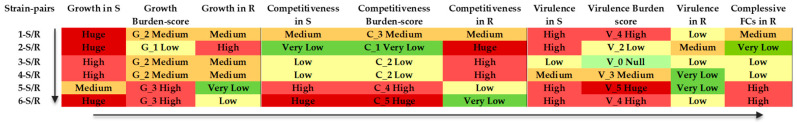
Bidimensional overview of FCs in growth, competitiveness, and virulence.

A bidimensional overview of growth, competitiveness, virulence FC burden, and their outputs in R strain, intra-strain pair (↓) and inter-strain pairs (→), is shown in Figure 5.

## 3. Discussion

The resistance-fitness/virulence interplay represents a significant concern in ESKAPE superbugs, such as MRSA, due to their considerable adaptability, which is associated with numerous virulence factors and antimicrobial resistance mechanisms. 

Deciphering the intricate blueprint of resistance-virulence co-evolution in the community or clinics could significantly impact the reduction of the epidemiological challenges associated with the management of healthcare-associated infections.

First- and second-line antimicrobials, such as glycopeptides and daptomycin, target essential cellular functions, such as peptidoglycan biosynthesis, cell membrane integrity, and lysis [22,23]; thus, the acquisition of resistance or reduced susceptibility can interfere with the occurrence of FCs, directly or indirectly affecting growth, competitiveness, and virulence.

The impact on essential biological functions strongly conditions the likelihood of the spread of determinate antimicrobial resistance or reduced susceptibility in specific settings (healthcare/hospitals or community settings) [18,24,25]. The trade-off among the growth-performance, competitiveness, and virulence is pivotal for maintaining the best possible fitness for a given environment. Growth performance reflects the metabolic functions implicated in the ability to survive antimicrobials; competitiveness influences the pathogen onset, spread, and transmissibility rates; and virulence determines the ability rate to cause a host disease.

Crucial considerations emerged in our investigation about the FC burden acquired in clinical and reference isogenic MRSA strain pairs of predominant high-risk MRSA clones, acquiring stepwise daptomycin and/or glycopeptide (vancomycin/teicoplanin/telavancin and dalbavancin) reduced susceptibility. A major likelihood of onset and spread of resistant lineages could be putatively predicted for DAP-R GSSA and DAP-R/S hGISA, having low FC burden, in free-antimicrobial settings as well as for DAP-R hGISA and GISA, paying the highest one but acquiring resistance, in antimicrobial high-pressure settings.

Analyzing the FC burden from a comparative point of view, FCs gradually appeared in DAP-R GSSA and DAP-S hGISA, followed by DAP-R hGISA and DAP-R GISA, even though their relative impact remains significantly high in each level of resistance. This suggested that all resistant strains—acquiring an extensively reduced susceptibility—can be fitter than their susceptible counterparts to overcome the selective antimicrobial pressure and to survive in high antimicrobial exposure settings. On the contrary, in free antimicrobial settings, all resistant strains—having lower fitness and higher fitness costs—could be outcompeted by their susceptible due to a lower potentiality to survive.

First, DAP-R GSSA acquired, comparatively, a low reduction in virulence, growth performance, and competitiveness. This is in a predominant HA-MRSA N315 lineage, ST-5, spatype-t2, agr-II, and SCCmecII lineage with a large virulome (Virulome_score: +3/3), in-vitro high virulence (strong staphyloxanthin and colony spreader, weak biofilm, and alpha-hemolysin producer) (in-vitro V_score: +8/10), along with a high in-vivo *G. mellonella* larvae killing ability (K_score: +6/7).

DAP-R GSSA onset determined the FCs—poorly but simultaneously—affecting growth, competitiveness, and virulence. FCs compromised growth ability by a small slowdown in lag-phase (2 h instead 1 h) (G_score: +1/3), competitiveness by a small-scale and short-term outcompeting of DAP-S vs. the DAP-R strain (C_ score: +1/5), and moderate virulence by a weak in-vitro decrease of staphyloxanthin production and medium colony-spreading ability (in-vitro V_reduction_score: +3/7), as well as having no changes in the in-vivo *G. mellonella* larvae-killing ability (in-vivo V_reduction_score: 0/5). These observations agreed with other previous findings [26].

Looking in-depth, DAP-R GSSA could be still a moderate-fitting microorganism versus their susceptible counterpart both in free antimicrobial settings—such as in the community—for paying only small-scale fitness costs and in DAP (and not in GLY) high-pressure environments—such as healthcare settings and hospitals – due to its DAP-resistance acquisition. This could support the low rate of global DAP-R occurrence, in agreement with other authors [27,28,29].

Second, hGISA acquired a comparatively high decreased virulence, medium-reduced competitiveness, and low growth slowdown. This occurred predominantly in HA-MRSA N315 RefGen, ST-5, spatype-2, agr-II, and SCCmecIIa lineage, having a large virulome (Virulome_score: +3/3) and high intrinsic virulence based on a medium in-vitro virulence (strong biofilm production, moderate staphyloxanthin and colony-spreading ability) (in-vitro V_score: +7/10), along with a huge in-vivo killing ability in a *G. mellonella* larvae model (K_score: +7/7). These findings were in agreement with Peleg et al. (2009) [30].

hGISA acquisition determined FCs highly impacting virulence, moderately impacting competitiveness, and weakly impacting growth. FCs determined DAP-S GSSA long-term and medium-scale outcompeting vs. DAP-S hGISA (C_score: +3/5) and slightly increased the lag-phase by 1 hr and the exponential doubling time after 2 h (G_score: +1/3). FCs highly affected virulence, determining a significant in-vitro virulence reduction (decrease in biofilm formation and a low reduction in staphyloxanthin formation, and colony spreading) (in-vitro V_reduction_ score: +4/7), as well as a medium in-vivo reduction in *G. mellonella* larvae killing ability (in-vivo V_reduction_score: +1/5).

The exclusive hGISA emergence—paying relatively moderate FCs—generated a moderately competitive, poorly slow-growing, and virulent microorganism containing a subset of the bacterial population with reduced susceptibility versus the “old glycopeptides” such as vancomycin/teicoplanin and telavancin. These findings demonstrated that in free-antimicrobial settings, hGISA DAP-S should be less fit versus susceptible strains, mainly for reduced-growth, competitiveness, and virulence. In glycopeptide high-pressure settings, hGISA acquired a better fitness for its ability to survive glycopeptide exposure by the occurrence of a resistant subpopulation.

Third, DAP-R hGISA comparatively acquired a medium decrease in growth performance and virulence in a lineage/strain-dependent manner as well as a low reduction in competitiveness.

DAP-R hGISA were an LA-MRSA ST-398 lineage, spatype-t1939, agr-I, SCCmecIVa, and HA-EMRSA-15, USA 100, ST-22, spatype-t25, agr-I, SCCmecIV. In both lineages, DAP-R hGISA FCs were paid by a long-term and small-scale decrease in competitiveness (C_score: +2/5), medium growth slowdown [a longer lag-phase 2 h, exponential hourly doubling gTs (about 30 min)], and alterations in the stationary phase compared to the DAP-S strain (G_score: +2/3). Concerning virulence, a lineage/strain-dependent mechanism needed to be considered. In detail, the DAP-S GSSA LA-MRSA ST-398 lineage had a small virulome (Virulome_score: +1/3), a low innate virulence (weak alpha-hemolysin, protease, and medium colony spreading) (in-vitro V_score: +4/10) as well as a poor in- vivo *G. mellonella* larvae-killing ability (K_score: +3/7). No FCs were paid both in in-vitro virulence (in-vitro V_reduction_score: 0/7) and in in-vivo *G. mellonella* larvae-killing ability (in-vivo V_reduction_score: 0/5). Conversely, DAP-S GSSA HA-EMRSA-15 ST-22 had a similar small virulome (Virulome_score: +1/3), a medium constitutive virulence (moderate in-vitro colony spreading, weak alpha-hemolysis, staphyloxanthin, and protease) (in-vitro V_score: +5/10) as well as a high in-vivo *G. mellonella* larvae-killing ability (K_score: +6/7). 

Otherwise, FCs poorly affected in-vitro virulence, lacking only protease production and decreasing to weaken the colony spreading (in-vitro V_reduction_score: +2/7), and also had a very high impact on in-vivo *G. mellonella* larvae-killing ability (in-vivo V_reduction_score:: +5/5). These findings agreed with previous findings [30].

DAP-R hGISA cross-resistance resulted in a new FC layout, paying high-impacting FCs leading to a medium slow growing, medium/low lineage-dependent virulent, and highly competitive microorganisms acquiring both DAP-RS and GLY heteroresistance. Under DAP and high pressure, DAP-R hGISA could be more fit for its partial cross-ability to survive DAP and GLY exposure, as known the most used antimicrobials as last-resort and first-line MRSA antimicrobials in clinical practice. On the contrary, DAP-R hGISA could be less fit versus susceptible strains for its intermediate slow growth and low inter-bacterial outcompeting in free-antimicrobial settings.

Lastly, DAP-R GISA comparatively acquired a simultaneously huge or high decrease in competitiveness, growth performance, and virulence in a lineage/strain-dependent manner.

This was recorded in a CA-MRSA MW2, ST-1, spatype-t127, agr-III, SCCmecIVa lineage, and in a HA-MRSA N315, ST-5, spatype-2, agr-II, SCCmecIIa lineage.

The CA-MRSA MW2 lineage had an innate medium virulome (Virulome_score: +2/3) and a high virulence based on a huge in-vitro virulence (strong colony spreading, moderate staphyloxanthin formation, weak alpha/delta-hemolysis and protease activity) (in-vitro V_score: +9/10) associated with an intermediate in-vivo *G. mellonella* larvae-killing ability (K_score: +5/7). The DAP-R GISA HA-MRSA N315 lineage had a large virulome (Virulome_score: +3/3) and a high virulence based on high in-vitro virulence (strong biofilm production, moderate staphyloxanthin, medium colony spreading) (in-vitro V_score: +7/10), and huge *G. mellonella* larvae-killing ability (K_score: +7/7).

In DAP-R GISA, FCs had a drastically high impact on competitiveness with DAP-S GSSA long-term and large-scale outcompeting the DAP-R GISA strain (C_score: +4-5/5), and growth extending the lag-phase by 3 h (G_score: +3/3). In DAP-R GISA CA-MRSA MW2, high FCs in virulence were generated by an in-vitro lack of alpha/delta-hemolysin and protease activity, a strong reduction in colony spreading, and a low decrease in staphyloxanthin production (in-vitro V_reduction_ score: +7/7), associated with a medium in vivo *G. mellonella* larvae-killing reduction (in-vivo V_reduction_score: +3/5). Similarly, in the DAP-R GISA HA-MRSA N315 lineage, FCs conferred a low virulence caused by a high in-vitro virulence reduction due to a significant decrease in biofilm formation and a modest decrease in staphyloxanthin production and colony spreading (in-vitro V_reduction_score: +4/7), along with a low in-vivo virulence reduction due to a low decrease in in-vivo *G. mellonella* larvae-killing ability (in-vivo V_reduction_score: +1/5).

DAP-R GISA onset produced a new expanded FC layout, paying the highest FCs in a lineage-dependent manner, generating low/very-low competitive and virulent and highly slow-growing microorganisms acquiring daptomycin resistance, as well as a homogeneous glycopeptide-reduced susceptibility in the whole bacterial population. These findings lead us to consider that DAP-R GISA is the best-fitting microorganism in DAP and GLY high-pressure settings, versus its susceptible counterparts, whereas very limited onsetting and selective options should occur in free-drug environments.

In conclusion, virulence is the first factor to be involved already at lower levels of resistance, such as hGISA and DAP-R. Virulence is to be looked at as an accessory trait, not essential for vitality. As proof, virulence genes represent part of the accessory genome and not the core one. Having to sacrifice something, the first choice is decreasing virulence to leave an opportunity for antimicrobial resistance. A great pool of virulence factors as well as larvae host-killing ability can be affected. Among these, the biofilm, involved in adherence, increasing persistence on surfaces, materials, and food, as well as complicating their elimination and treatment [29]; hemolysins, involved in toxigenicity; staphyloxanthin, involved in the oxidative stress and ROS responses; proteases, involved in the degradation of host tissues and food; and Phenol-Soluble Modulins (PSMs), including PSMs-a, PSM-b, and *hld*, which are related to pathogenesis, such as the lysis of leukocytes and erythrocytes, stimulation of inflammatory responses, and contribution to biofilm development [31,32,33].

Competitiveness can be simultaneously targeted. This feature affects the outcompeting ability and, hence, to spread in the absence/presence of antimicrobials. The microorganism lowers the ability to outcompete, spread, and transmit itself at low antimicrobial pressure but it improves the possibility of surviving in settings with a high pressure of antimicrobials.

To acquire extensive antimicrobial resistance, FCs necessarily affect growth performance. Growth, unlike virulence, is an essential aspect of the microorganism’s life based on essential gene activity and, hence, the core genome. There is a current consensus that cells with slow metabolism or in dormancy underlie bacterial survival to antimicrobials [34,35]. A trade-off is established with a drastic growth slowdown in addition to sacrificing a reduction in virulence and competitiveness. Overall, DAP-R GISA drastically adapts itself to decrease its virulence, and thus its ability to cause diseases and transmissibility, acquiring an extensive resistance to face and overcome DAP and GLY exposure [11].

A second important aspect is that this behavior is supported by genomes prone to do so. The extensive degree of DAP- and GLY-RS emerges in genomes with a medium or large virulome, developing a high virulence. These can thus balance FCs with more accessory resources; before affecting the vital ones. This is an expedient to balance the genomic attitude of the different lineages. To acquire resistance, high virulent lineages decrease virulence factor production. This can be more useful in the sustaining of the antimicrobial resistance pathways versus the virulence ones.

In conclusion, our findings demonstrated that acquiring daptomycin and progressively extensive rates of reduced susceptibility to glycopeptides requires increasing FCs. These costs primarily compromise virulence, competitiveness, and growth performance, either by exploiting genomes with low pathogenic potential or by genomically and phenotypically reducing virulence. As glycopeptide resistance levels increase, stepwise alterations also occur in competitiveness and growth performance. This establishes a critical bridge connecting growth dynamics to pathogenicity.

## 4. Materials and Methods

### 4.1. Bacterial Strains

To investigate the fitness cost burden without bias due to similarity or diversity, a sample of clinical and reference isogenic strain pairs of “old and new”-circulating high-risk clones of epidemiologically different groups (HA-MRSA, LA-MRSA, CA-MRSA) and genomic backgrounds stepwise acquiring exclusive hGISA, DAP-RS, or GLY and DAP cross-resistance were included in this study.

All R strains, i.e., the DAP-R, hGISA, and/or GISA strains, emerged by S parents under DAP or GLY mono or combination therapy, as previously described [25,36].

To simplify the reduced-susceptibility, DAP-R, hGISA, and GISA strains were indicated as the R strain, whilst their susceptible parents were the S strain.

A clinical-reference DAP-S hGISA and DAP-S GSSA 1-R/S (MU3/N315) strain pair having VAN, TEC, and TEL hGISA phenotype [36]; a clinical DAP-R GSSA and DAP-S GSSA 2-R/S strain pair acquiring uniquely DAP-RS [37]; a clinical DAP-R hGISA/DAP-S GSSA 3-R/S strain pair with a DAP-RS and VAN, TEC, TEL hGISA phenotype [37]; a clinical DAP-R hGISA/DAP-S GSSA 4-R/S strain pair developing DAP-RS and VAN, TEC, and TEL hGISA phenotype [25]; a clinical DAP-R GISA/DAP-S GSSA 5-R/S strain pair acquiring DAP-RS and dalbavancin (DAL), VAN, TEC, and TEL GISA phenotype [18,24,25]; and clinical-reference DAP-R GISA/DAP-S GSSA 6-R/S (MU50/N315)—DAP-R and DAL, VAN, TEC, TEL GISA [36]—were included in this study. The AMR profiles are shown in Table S1.

### 4.2. Antimicrobial Susceptibility Assays

Antimicrobial susceptibility tests were assessed according to the EUCAST 2025 guidelines (European Committee on Antimicrobial Susceptibility Testing. Breakpoint tables for interpretation of MICs and diameter zone. Version 15.0, 2025. http://www.eucast.org, accessed on 4 February 2025). *S. aureus* ATCC 29213 was used as the control strain. Antibiotic susceptibility and genomic characterization were described previously [18,24].

### 4.3. Fitness Experiments

FCs were investigated as previously described [38], with the same modifications as briefly described. MRSA strains were grown on Brain Heart Infusion Agar (BHIA) (Oxoid), and five well-isolated colonies were inoculated into 20 mL of Brain Heart Infusion Broth (BHIB). The tubes were then kept in a water bath at 37 °C with shaking (80 rpm). After overnight incubation, cultures were diluted to an optical density of λ = 600 nm (OD_600_) 0.05 (stock solutions). All experiments were performed in three independent biological assays in triplicate for each strain culture.

#### 4.3.1. Independent Growth

For independent-growth experiments, 200 µL of single-strain solutions were added to 20 mL of fresh BHIB in a sterile 50 mL tube. The initial inoculum of both strains was approximately 1 × 10^5^ CFU/mL. OD_600_ readings and vital counts were performed every hour for 7 h, lastly at 24 h.

#### 4.3.2. Mixed Growth

For mixed-growth experiments, S and R MRSA of each couple sample stock solutions (200 µL) were added to the same 20 mL of fresh BHIB in a sterile 50 mL tube. The final inoculum at baseline was approximately 1 × 10^5^ CFU/mL. Appropriate dilutions were plated in triplicate onto selective vancomycin or daptomycin plates (at concentrations derived from MICs). Non-selective BHIA, every hour for 7 h, lastly at 24 h, was used for the growth of both the S and R samples. Viable counts on the selective plates provided the R growth levels while the difference between selective and non-selective plate colony counts provided the bacterial growth rate.

#### 4.3.3. Fitness Measures

Growth rates were calculated hourly between 1 and 7 h and at 24 h timepoints from independent-growth and mixed-growth samples using the following equation for growth rate (r):

$$r=\frac{ln\left(FinalPopulation\right)–ln\left(InitialPopulation\right)}{time}$$

where growth rate (r) is the rate at which the population is growing or declining per unit of time (usually expressed in units per hour). ln(x) represents the natural logarithm of x. The final population is the number of bacteria at the end of the time. The initial population is the number of bacteria at the beginning of the time. Time is the duration over which the bacterial growth occurred (typically measured in hours). The growth rate is usually expressed in units per hour (e.g., bacteria per hour). A positive growth rate indicates an increase in the bacterial population. A negative growth rate suggests a decrease in the population (e.g., in the case of bacterial decay). A growth rate of zero indicates no change in the population. Statistical analysis of growth rate was performed with the software GraphPad Prism version 9 using one-way analysis of variance (ANOVA) and 95% confidence intervals (CI 95%). Continuous variables were statistically analyzed with an independent *t*-test. *p* < 0.05 was statistically significant.

#### 4.3.4. In-Vitro Biofilm Formation

Biofilm assays were carried out as described previously [39].

#### 4.3.5. In-Vitro Hemolysin, Staphyloxanthin, and Caseinase Production

To determine hemolysin, staphyloxanthin, and caseinase production, assays were performed as previously described [37]. Briefly, 5 μL of overnight culture from each strain was inoculated onto Tryptone Soy Agar (TSA) plates and incubated at 37 °C for 20–24 h to assess staphyloxanthin synthesis. Staphyloxanthin production was evaluated by observing the development of an orange-yellow color. For hemolysin production, 5 μL of overnight culture from each strain was placed on blood agar plates and incubated at 37 °C for 24 h. The activity of alpha-hemolysin was determined by the presence or absence of a clear zone around the colonies. After another incubation at 4 °C for 24 h, the plates were observed again to assess the effects of beta hemolysin. Delta hemolysin was assessed as previously described [40]. The caseinase activity was assessed by inoculating 5 μL of overnight culture from each strain onto plates containing nutrient broth (Becton, Dickinson and Company, Sparks, NV, USA) supplemented with 1.5% agar and 1.5% skim milk (OXOID, Hampshire, England). After 24 h of incubation at 37 °C, a clear zone around the bacterial colonies indicated positive proteolytic activity.

#### 4.3.6. Colony Spreading Ability

An amount of 2 μL of overnight culture from each strain was inoculated onto Tryptone Soy Broth (TSB) medium containing 0.25% agar and incubated overnight at 37 °C. Following incubation, the diameter of each bacterial colony was measured (size of colonies) [41].

#### 4.3.7. Statistical Analysis

All experiments were carried out in triplicate. Comparisons between different sample groups were made using a one-way analysis of variance (ANOVA) and a post hoc Tukey HSD test.

#### 4.3.8. In-Vivo *G. mellonella* Infection Assay

The virulence of each strain pair was evaluated by the in-vivo model of *G. mellonella* larvae, representing a recognized model that does not require ethical approval. Strains were cultivated since 10^8^ CFU/mL in BHIB. Three groups of five randomly selected *G. mellonella* larvae were injected with 10 μL of MRSA broth culture in the last left proleg of each larva. *G. mellonella* larvae were incubated at 37 °C in the dark for 6 days. A group of five larvae had no infection (untreated group). A control group of five larvae was treated only with 10 μL PBS (control group) and the other five larvae only received a sham injection (sham injection group; larvae were punctured with a sterile syringe to evaluate the effect of the larval puncture). During assays, larvae did not receive nutrition.

The *G. mellonella* survival was controlled every day. The dead larvae were unresponsive to touch and black-colored. Data were obtained from at least three independent experiments performed in triplicate.

### 4.4. Whole-Genome Sequencing 

#### 4.4.1. DNA Extraction

Following the manufacturer’s instructions, genomic DNA was extracted using the PureLink Genomic DNA Mini Kit (Invitrogen, Waltham, MA, USA) as previously published [18,24,37].

#### 4.4.2. Whole-Genome Sequencing (WGS)

Whole-genome sequencing (WGS) was performed with an Illumina MiSeq system using paired-end (PE) read libraries prepared with the Nextera XT DNA Library Prep Kit (Illumina, San Diego, CA, USA). The libraries were evaluated using published methods [42]. The raw reads were assessed using FastQC (v.0.11.7). The Cutadapter tool (v.1.16), implemented in Python (v.3.5.2), was used to remove residual PCR primers and filter low-quality bases (Q_score < 30) and short reads (<150 bp) [42]. The filtered trimmed reads were included in the downstream analysis.

### 4.5. De Novo Genome Assembly

Using the SPAdes program (v3.12.0), a de novo genome assembly was carried out. Each sample was given a contigs file by the SPAdes program, and post-assembly controls and metrics were assessed using Quast software (v4.6.3), as previously published [18,24,37].

### 4.6. Gene Annotation

Prokka software (1.14.6) was used to analyze the assembled contigs to predict genes and to annotate using a core set of conserved prokaryotic genes, as previously published [18,24,37]. 

### 4.7. Analysis of Mobile Genetic Elements

The Prokka -annotated genomes of all the strains were used to screen genomic islands with the IslandViewer 4 tool (https://www.pathogenomics.sfu.ca/islandviewer, accessed on 4 February 2025) [43]. MGE finder and PlasmidFinder 2.1 were used to perform the detection of MGE and plasmids (https://cge.food.dtu.dk/services/PlasmidFinder/, accessed on 4 February 2025) [44], respectively. For *scc*-mec typing, SCCmecFinder1.2 was used with the following parameters: 90% minimum identity and 60% minimum coverage (https://cge.food.dtu.dk/services/SCCmecFinder/, accessed on 4 February 2025) [45].

### 4.8. Reference Genome Similarity

A single-nucleotide polymorphism (SNP) tree was constructed using CSI Phylogeny 1.4 (https://cge.food.dtu.dk/services/CSIPhylogeny/, accessed on 4 February 2025). This analysis was performed using the default setting, excluding heterozygous SNPs. The following criteria for high-quality SNP calling and filtering were selected: (i) a minimum depth of 10× at SNP positions, (ii) a minimum relative depth of 10% at SNP positions, (iii) a minimum distance of 10 bp between SNPs, (iv) a minimum SNP quality of 30, (v) a minimum read mapping quality of 25, and (vi) a minimum Z score of 1.96 [46].

### 4.9. Typing and Identification of Virulence and Antimicrobial Resistance Genes

To determine the sequence type (ST) of the isolated strains, in silico MLST analysis was performed using the MLST 2.0 database (https://cge.food.dtu.dk/services/MLST/, accessed on 4 February 2025) [47]. The VBDF Virulence website was used to define virulomes (https://www.mgc.ac.cn/cgi-bin/VFs/v5/main.cgi, accessed on 4 February 2025) [48].

### 4.10. Spa Types

Spa types were predicted using spaTyper v1.0 webserver from the Center of Genomic Epidemiology (https://cge.food.dtu.dk/services/spaTyper/, accessed on 4 February 2025) [49].

### 4.11. Genome Accession Numbers

Eight *S. aureus* genomic reads have been deposited in the National Center for Biotechnology Information (NCBI) SRA database under SUB15044456 with BioProject accession number PRJNA1216545 (2-S/R,4-S/R), SUB11823107 (5-S/R) with BioProject accession number PRJNA860577, SRP166981 with the BioProject accession number PRJNA498510(3-S/R). The sequences of strains MW2, N315 (1-S, 6-S), Mu3 (1-R), and Mu50 (6-R) were available in public databases with accession numbers NC_003923.1, NC_002745.2, NC_009782.1, NC_002758.2, respectively.

## Figures and Tables

**Table 1:** In-vitro virulence factor analysis.

	1-S	1-R	2-S	2-R	3-S	3-R	4-S	4-R	5-S	5-R	6-S	6-R	USA 300	MW2
Biofilm	+++	+	+	+	−	−	−	−	−	−	+++	+	+	++
α-hemolysis	−	−	+	+	+	+	+	+	+	−	−	−	+	+
β-hemolysis	−	−	−	−	−	−	−	−	−	−	−	−	−	−
δ-hemolysis	−	−	−	−	−	−	−	−	+	−	−	−	+	+
Staphyloxanthin	++	+	+++	+	-	-	+	+	++	+	++	+	+	++
Protease activity	−	−	−	−	+	+	+	−	+	−	−	−	+	+
Colony spreading	++	+	+++	++	++	++	++	+	++++	+	++	+	++	+++

Legend: − negative; + weak positive; ++ medium positive; +++ strong positive; ++++ high strong positive.

**Table 2:** In-vivo *G. mellonella* killing assay.

Strains	Killing Ability										K_Score
	24 h		48 h		72 h		96 h		120 h		
	S	D	S	D	S	D	S	D	S	D	
1-S	0	5	-	-	-	-	-	-	-	-	+7
1-R	1	4	0	5	-	-	-	-	-	-	+6
2-S	3	2	0	5	-	-	-	-	-	-	+6
2-R	4	1	0	5	-	-	-	-	-	-	+6
3-S	2	3	1	4	1	4	1	4	1	4	+3
3-R	1	4	1	4	1	4	1	4	1	4	+3
4-S	2	3	0	5	-	-	-	-	-	-	+6
4-R	4	1	3	2	3	2	3	2	3	2	+1
5-S	3	2	1	4	0	5	-	-	-	-	+5
5-R	4	1	3	2	3	2	2	3	2	3	+2
6-S	0	5	-	-	-	-	-	-	-	-	+7
6-R	1	4	0	5	-	-	-	-	-	-	+6
MW2	1	4	0	5	-	-	-	-	-	-	+6
USA300	4	1	1	4	1	4	0	5	-	-	+4
Control group	5	0	5	0	5	0	5	0	5	0	0
Untreated group	5	0	5	0	5	0	5	0	5	0	0
Sham injection group	5	0	5	0	5	0	5	0	5	0	0

Legend: S: surviving, D: dead

## Data Availability

The genomic reads were deposited in the National Center for Biotechnology Information (NCBI) Genome database in the Sequence Read Archive (SRA). Eight S. aureus genomic reads have been deposited in the National Center for Biotechnology Information (NCBI) SRA database under SUB15044456 with BioProject accession number PRJNA1216545 (2-S/R,4-S/R), SUB11823107 (5-S/R) with BioProject accession number PRJNA860577, SRP166981 with BioProject accession number PRJNA498510(3-S/R). The sequences of strains MW2, N315 (1-S, 6-S), Mu3 (1-R), and Mu50 (6-R) are available in public databases with accession numbers NC_003923.1, NC_002745.2, NC_009782.1, NC_002758.2, respectively.

## Supplementary Materials

The following supporting information can be downloaded at: https://www.mdpi.com/article/10.3390/antibiotics14030244/s1, Table S1: Isogenic strain-pairs, AMR Profiles and Genomic Typing; Table S2: Mobile Genetic Elements, Resistome and SNPs related to AMR phenotype; Table S3: Virulence factors; Table S4: Generation times and Growth-rates of MRSA couples; Table S5: Competitive growth of MRSA couples; Table S6: in-vitro virulence factor analysis data; Figure S1: α-hemolysin activity assay; Figure S2: Staphyloxanthin production; Figure S3: Caseinase production.
