# Supplementary material for: Fitness Burden for the Stepwise Acquisition of First- and Second-Line Antimicrobial Reduced-Susceptibility in High-Risk ESKAPE MRSA Superbugs
Source: Antibiotics (Basel). 2025 Feb 28;14(3):244. doi: 10.3390/antibiotics14030244 (PMC11939686; doi:10.3390/antibiotics14030244)
Supplement: Supplementary file 1 [file antibiotics-14-00244-s001.zip › antibiotics-3486475-Table S1.pdf]

Table.S1: Isogenic strain-pairs, AMR Profiles and Genomic Typing

ISOGENIC STRAIN-PAIRS	AMR PROFILES						GENOMIC TYPING				
	VAN MICs (mg/L)	TEC MICs (mg/L)	DAL MICs (mg/L)	TLV MICs (mg/L)	DAP MICs (mg/L)	DAP/GLY PAP-ANALYSIS	CSI Phylogeny RefGen Similarity	MLST	spa-type	scc-mec	agr-group
**1-S**	0,5	0,25	0,064	0,047	0,25	DAP-S GSSA	N315 HA-MRSA	ST-5	t2	IIa	II
**1-R**	2	8	0,38	0,094	1	DAP-S hGISA					
**2-S**	1	2	0,047	0,094	0,5	DAP-S GSSA	N315 HA-MRSA	ST-5	t2	II	II
**2-R**	2	2	0,047	0,19	2	DAP-R GSSA					
**3-S**	1	<0,25	0,023	0,125	<0,25	DAP-S GSSA	ST398 LA-MRSA	ST-398	t1939	IVa	I
**3-R**	2	2	0,064	0,19	4	DAP-R hGISA					
**4-S**	1	0.5	0.047	0,19	<0.25	DAP-S GSSA	USA 100 HA-EMRSA15	ST-22	t25	IV	I
**4-R**	1	1	0.016	0,19	2	DAP-R hGISA					
**5-S**	1	1	0,064	0,19	0,5	DAP-S GSSA	USA400 CA-MW2-MRSA	ST-1	t127	IVa	III
**5-R**	8	32	2	0,19	2	DAP-R GISA					
**6-S**	0,5	0,25	0,064	0,047	0,25	DAP-S GSSA	N315 HA-MRSA	ST-5	t2	IIa	II
**6-R**	8	16	0,75	0,125	2	DAP-R GISA					

Legend: VAN: vancomycin; TEC: teicoplanin; DAL: dalbavancin; TLV: telavancin; DAP: daptomycin; GLY: glycopeptides
