# Supplementary material for: Fitness Burden for the Stepwise Acquisition of First- and Second-Line Antimicrobial Reduced-Susceptibility in High-Risk ESKAPE MRSA Superbugs
Source: Antibiotics (Basel). 2025 Feb 28;14(3):244. doi: 10.3390/antibiotics14030244 (PMC11939686; doi:10.3390/antibiotics14030244)
Supplement: Supplementary file 1 [file antibiotics-14-00244-s001.zip › antibiotics-3486475-Table S2.pdf]

Table.S2: Mobile Genetic Elements, Resistome and SNPs related to AMR phenotype

	Plasmid	Transposon	Insertion sequence	Genomic Islands (GIs)	Resistome	AMR SNPs
**1-S**<br>DAP-S GSSA	rep22	Tn554	ISSau4	12	Aminoglycoside: *aadD, ant(9)-Ia*<br>Beta-lactams: *mecA*<br>Glycopeptides: *bleO*<br>Macrolide/ Lincosamide: *erm(A)*	-
**1-R**<br>DAP-S hGISA	rep22	Tn554	ISSau4<br>ISLgar5	11	Aminoglycoside: *aadD, ant(9)-Ia*<br>Beta-lactams: *mecA*<br>Glycopeptides: *bleO*<br>Macrolide/ Lincosamide: *erm(A)*<br>Tetracycline: *tet(M)*	*grlA:* p.S80F (ciprofloxacin)<br>*gyrA:* p.S84L (ciprofloxacin)
**2-S/R**<br>DAP-S GSSA/DAP-R GSSA	rep20<br>rep22	Tn554	ISSau4	10/3	Aminoglycoside: *aadD, ant(9)-Ia*<br>Beta-lactams: *mecA, blaZ*<br>Glycopeptides: *bleO*<br>Macrolide/ Lincosamide: *erm(A)*	*grlA:* p.S80Y (ciprofloxacin)<br>*gyrA:* p.S84L (ciprofloxacin)<br>*rpoB:* p.A477D (rifampicin)
**3-S/R**<br>DAP-S GSSA/DAP-R hGISA	rep10<br>repUS43<br>rep15	Tn6009	ISSau8	9/9	Aminoglycoside: *aac(6')- aph(2'')*,<br>Beta-lactams: *mecA*,<br>Fosfomycin: *fosD*<br>Macrolide/ Lincosamide: *erm(C), vga(A)V, fexB*<br>Tetracycline: *tet(M)*	*grlA:* p.S80F (ciprofloxacin)<br>*rpoB:* p.A477D (rifampicin)
**4-S/R**<br>DAP-S GSSA/DAP-R hGISA	rep5A<br>rep10<br>rep20	-	ISSau2<br>ISSau5<br>ISSau6	7/7	Beta-lactams: *mecA, blaZ*<br>Macrolide/ Lincosamide: *erm(C)*	grlA: p.S80F (ciprofloxacin)<br>gyrA: p.S84L (ciprofloxacin)
**5-S/R**<br>DAP-S GSSA/ DAP-R GISA	rep7a<br>rep5a<br>rep16<br>rep10<br>rep7c	-	-	12/6	Aminoglycoside: *ant(6)-Ia, aph(3')-III*<br>Beta-lactams: *blaz, mecA*<br>Macrolide/ Lincosamide:*erm(C)* Tetracycline: *tet(K)*	*grlA:* p.S80F (ciprofloxacin)<br>*gyrA:* p.S84L (ciprofloxacin)
**6-R**<br>DAP-R GISA	rep22	Tn554	ISSau4<br>ISLgar5	12	Aminoglycoside: *aadD, ant(9)-Ia*<br>Beta-lactams: *mecA*<br>Glycopeptides: *bleO*<br>Macrolide/ Lincosamide: *erm(A)*<br>Tetracycline: *tet(M)*	*grlA:* p.S80F (ciprofloxacin)<br>*gyrA:* p.S84L (ciprofloxacin)
