# Supplementary material for: Fitness Burden for the Stepwise Acquisition of First- and Second-Line Antimicrobial Reduced-Susceptibility in High-Risk ESKAPE MRSA Superbugs
Source: Antibiotics (Basel). 2025 Feb 28;14(3):244. doi: 10.3390/antibiotics14030244 (PMC11939686; doi:10.3390/antibiotics14030244)
Supplement: Supplementary file 1 [file antibiotics-14-00244-s001.zip › antibiotics-3486475-Table S3.pdf]

Table.S3: Virulence factors

Virulence factors	Related genes	1/6-S S. aureus subsp. aureus N315 chromosome NC_002745	1-R S. aureus subsp. aureus Mu3 chromosome NC_009782	2-S/R	3-S/R	4-S/R	5-S/R	6-R S. aureus subsp. aureus Mu50 chromosome NC_002758	S. aureus subsp. aureus MW2 chromosome NC_003923	S. aureus subsp. aureus USA300_FPR3757 chromosome NC_007793
Adherence	**14 genes**	**9 genes**	**10 genes**	**9 genes**	**10 genes**	**8 genes**	**9 genes**	**9 genes**	**11 genes**	**9 genes**
**Autolysin alt**	*atl*	SA_RS05135	SAHV_RS05545	CCIJOALM_00906	orf00442	MMEFEJFM_02048	JCKDIPHL_01344	SAV_RS05680	MW_RS05030	SAUSA300_RS05135
**Clumping factor**	*clfA*	SA_RS04225	SAHV_RS04305	CCIJOALM_00337	orf00282				MW_RS04155	SAUSA300_RS04165
	clfB	SA_RS13895	SAHV_RS14235	CCIJOALM_00674			JCKDIPHL_00552	SAV_RS14375	MW_RS13850	SAUSA300_RS14270
**CNA**	*cna*				orf00319	MMEFEJFM_02162	JCKDIPHL_00614		MW_RS14170	
**Eap/Map**	*eap/map*		SAHV_RS10480*		orf01758	MMEFEJFM_01509		SAV_RS10615*	MW_RS10240*	
**EbpS**	*ebp*	SA_RS07440	SAHV_RS07850	CCIJOALM_00646	orf00856	MMEFEJFM_01045	JCKDIPHL_01070	SAV_RS07985	MW_RS07360	SAUSA300_RS07485
**FnBPs**	*fnbA*	SA_RS13135	SAHV_RS13510	CCIJOALM_01898	orf00129	MMEFEJFM_02007	JCKDIPHL_00423	SAV_RS13650	MW_RS13135	SAUSA300_RS13530
	fnbB	SA_RS13130	SAHV_RS13505	CCIJOALM_01897	orf00126; orf00127		JCKDIPHL_00422	SAV_RS13645	MW_RS13130	SAUSA300_RS13525
**SDr**	*sdrC*	SA_RS03025	SAHV_RS02995	CCIJOALM_01335	orf02259	MMEFEJFM_00045	JCKDIPHL_01605	SAV_RS03130	MW_RS02840	SAUSA300_RS02915
	sdrD	SA_RS03030	SAHV_RS03000	CCIJOALM_01336	orf02260	MMEFEJFM_00046	JCKDIPHL_01606	SAV_RS03135	MW_RS02845	SAUSA300_RS02920
	sdrE	SA_RS03035	SAHV_RS03005	CCIJOALM_01337	orf01042	MMEFEJFM_00047	JCKDIPHL_01607	SAV_RS03140	MW_RS02850	SAUSA300_RS02925
	sdrF									
	sdrG									
	sdrH									
Effector delivery system	**12 genes**	**12 genes**	**12 genes**	**12 genes**	**6 genes**	**7 genes**	**12 genes**	**12 genes**	**12 genes**	**12 genes**
**Type VII secretion system**	*esxA*	SA_RS01590	SAHV_RS01445	CCIJOALM_00171	orf01372	MMEFEJFM_01250	JCKDIPHL_02243	SAV_RS01590	MW_RS01365	SAUSA300_RS01490
	esaA	SA_RS01595	SAHV_RS01450	CCIJOALM_00170	orf01371	MMEFEJFM_01249	JCKDIPHL_02242	SAV_RS01595	MW_RS01370	SAUSA300_RS01495
	essA	SA_RS01600	SAHV_RS01455	CCIJOALM_00169	orf01370	MMEFEJFM_01248	JCKDIPHL_02241	SAV_RS01600	MW_RS01375	SAUSA300_RS01500
	esaB	SA_RS01605	SAHV_RS01460	CCIJOALM_00168		MMEFEJFM_01247	JCKDIPHL_02240	SAV_RS01605	MW_RS01380	SAUSA300_RS01505
	essB	SA_RS01610	SAHV_RS01465	CCIJOALM_00167	orf01368	MMEFEJFM_01246	JCKDIPHL_02239	SAV_RS01610	MW_RS01385	SAUSA300_RS01510
	essC	SA_RS01615	SAHV_RS01470	CCIJOALM_00166	orf01367	MMEFEJFM_01245	JCKDIPHL_02238	SAV_RS01615	MW_RS01390	SAUSA300_RS01515
	esxC	SA_RS01620	SAHV_RS01475	CCIJOALM_00165			JCKDIPHL_02237	SAV_RS01620	MW_RS01395	SAUSA300_RS01520
	esxB	SA_RS01625	SAHV_RS01480	CCIJOALM_00164			JCKDIPHL_02236	SAV_RS01625	MW_RS01400	SAUSA300_RS01525
	esaE	SA_RS01630	SAHV_RS01485	CCIJOALM_00163			JCKDIPHL_02235	SAV_RS01630	MW_RS01405	SAUSA300_RS01530
	esxD	SA_RS01635	SAHV_RS01490	CCIJOALM_00162			JCKDIPHL_02234	SAV_RS01635	MW_RS01410	SAUSA300_RS01535
	esaD	SA_RS01640	SAHV_RS01495	CCIJOALM_00160			JCKDIPHL_02233	SAV_RS01640	MW_RS01415	SAUSA300_RS01540
	esaG	SA_RS01685, SA_RS01680, SA_RS01675, SA_RS01670, SA_RS01665, SA_RS01645	SAHV_RS01540, SAHV_RS01535, SAHV_RS01530, SAHV_RS01525, SAHV_RS01520, SAHV_RS01500	CCIJOALM_00152; CCIJOALM_00153; CCIJOALM_00154; CCIJOALM_00155;	orf01358; orf01359	MMEFEJFM_01621;MMEFEJFM_01622	JCKDIPHL_02226; JCKDIPHL_02227; JCKDIPHL_02228; JCKDIPHL_02229;	SAV_RS01685, SAV_RS01680, SAV_RS01675, SAV_RS01670, SAV_RS01665, SAV_RS01645	MW_RS01470, MW_RS01465, MW_RS01460, MW_RS01455, MW_RS01450, MW_RS01440, MW_RS01435, MW_RS01445, MW_RS01420	SAUSA300_RS01605, SAUSA300_RS01610, SAUSA300_RS01600, SAUSA300_RS01595, SAUSA300_RS01

				CCIJOALM_00159			JCKDIPHL_02232			590, SAUSA300_RS01580, SAUSA300_RS01575*, SAUSA300_RS01570, SAUSA300_RS01565, SAUSA300_RS01545
Exotoxin	**67 genes**	**30 genes**	**30 genes**	**25 genes**	**14 genes**	**20 genes**	**19 genes**	**29 genes**	**28 genes**	**24 genes**
**hly/hla**	*hly/hla*	SA_RS05730	SAHV_RS06140	CCIJOALM_02655	orf00545	MMEFEJFM_01773	JCKDIPHL_01516	SAV_RS06275	MW_RS05625	SAUSA300_RS05720
	hlb	SA_RS10405*	SAHV_RS10815*					SAV_RS10950*	MW_RS10565*	SAUSA300_RS10840*
**hld**	*hld*	SA_RS10565	SAHV_RS10975	CCIJOALM_00756	orf01771	MMEFEJFM_01593	JCKDIPHL_02131	SAV_RS11110	MW_RS10655	SAUSA300_RS10930
	hlgA	SA_RS12670	SAHV_RS13050	CCIJOALM_02064; CCIJOALM_02320	orf00050	MMEFEJFM_00972	JCKDIPHL_00346; JCKDIPHL_02382	SAV_RS13185	MW_RS12690	SAUSA300_RS13070
**hlgB**	*hlgC*	SA_RS12675	SAHV_RS13055	CCIJOALM_01437	orf00051	MMEFEJFM_00973	JCKDIPHL_00347	SAV_RS13190	MW_RS12695	SAUSA300_RS13075
	hlgB	SA_RS12680	SAHV_RS13060	CCIJOALM_01438	orf00052	MMEFEJFM_00974	JCKDIPHL_00348	SAV_RS13195	MW_RS12700	SAUSA300_RS13080
**LukED**	*lukD*	SA_RS09210	SAHV_RS09630	CCIJOALM_02319			JCKDIPHL_02383	SAV_RS09765	MW_RS09430	SAUSA300_RS09680
	lukE	SA_RS09215	SAHV_RS09635					SAV_RS09770	MW_RS09435	SAUSA300_RS09685
**PVL**	*lukSPV*								MW_RS07415	SAUSA300_RS07545
	lukFPV								MW_RS07410	SAUSA300_RS07540
	lukM lukFlike									
**SE**	*sea*		SAHV_RS10540	CCIJOALM_02163				SAV_RS10675	MW_RS10305	
	seb									
	sec	SA_RS10435	SAHV_RS10845					SAV_RS10980	MW_RS04125	
	seg	SA_RS09240	SAHV_RS09660	CCIJOALM_02162; CCIJOALM_02572		MMEFEJFM_02430		SAV_RS09795		
	seh						JCKDIPHL_02096		MW_RS00265	
	sei	SA_RS09260	SAHV_RS09680			MMEFEJFM_02433		SAV_RS09815		
	selk			CCIJOALM_02568					MW_RS10555	SAUSA300_RS04320
	sell	SA_RS10430	SAHV_RS10840					SAV_RS10975	MW_RS04130	
	selm	SA_RS09265	SAHV_RS09685	CCIJOALM_02567		MMEFEJFM_02434		SAV_RS09820		
	seln	SA_RS09245	SAHV_RS09665	CCIJOALM_02571		MMEFEJFM_02431		SAV_RS09800		
	selo	SA_RS09270	SAHV_RS09690	CCIJOALM_02554		MMEFEJFM_02435		SAV_RS09825		
	selp	SA_RS10125								
	selq								MW_RS10550	SAUSA300_RS04325
	selu					MMEFEJFM_02432				
	yent1	SA1645	SAHV_1812	CCIJOALM_02569				SAV1827		
	yent2	SA1644	SAHV_1811	CCIJOALM_02570				SAV1826		
**SpA**	*spa*	SA_RS00690	SAHV_RS00545	CCIJOALM_02062	orf01847	MMEFEJFM_01413	JCKDIPHL_02284	SAV_RS00690	MW_RS00440	SAUSA300_RS00585

**SSLs**	*set-01*				orf01240	MMEFEJFM_01722				
	set-02				orf01243; orf01244	MMEFEJFM_01720				
	set-03				orf01241					
	set-04				orf01238	MMEFEJFM_01724				
	set-05				orf01239					
	set-06	SA_RS02185	SAHV_RS02165	CCIJOALM_00059	orf01246			SAV_RS02305		
	set-07	SA_RS02190	SAHV_RS02170	CCIJOALM_00058	orf01245	MMEFEJFM_01719		SAV_RS02310		
	set-08	SA_RS02195	SAHV_RS02175	CCIJOALM_00057				SAV_RS02315		
	set-09	SA_RS02205	SAHV_RS02185	CCIJOALM_00056						
	set-10	SA_RS02210	SAHV_RS02190	CCIJOALM_00055		MMEFEJFM_01721		SAV_RS02325		
	set-11	SA_RS02220	SAHV_RS02200	CCIJOALM_00054				SAV_RS02335		
	set-12	SA_RS02225	SAHV_RS02205	CCIJOALM_00053				SAV_RS02340		
	set-13	SA_RS02230	SAHV_RS02210	CCIJOALM_00052		MMEFEJFM_01723		SAV_RS02345		
	set-14	SA_RS02235	SAHV_RS02215					SAV_RS02350		
	set-15	SA_RS02255	SAHV_RS02235	CCIJOALM_00048		MMEFEJFM_01727		SAV_RS02370		
	set-16					MMEFEJFM_01718	JCKDIPHL_00753		MW_RS02010	
	set-17						JCKDIPHL_00754		MW_RS02015	
	set-18						JCKDIPHL_00755		MW_RS02020	
	set-19						JCKDIPHL_00756		MW_RS02030	
	set-20						JCKDIPHL_00757		MW_RS02035	
	set-21						JCKDIPHL_00758		MW_RS02045	
	set-22						JCKDIPHL_00759		MW_RS02050	
	set-23						JCKDIPHL_00760		MW_RS02055	
	set-24						JCKDIPHL_00761		MW_RS02060	
	set-25						JCKDIPHL_00762		MW_RS02065	
	set-26				orf02305		JCKDIPHL_02652		MW_RS02085	
	set-30									SAUSA300_RS02110
	set-31									SAUSA300_RS02115
	set-32									SAUSA300_RS02120
	set-33									SAUSA300_RS02130
	set-34									SAUSA300_RS02135
	set-35									SAUSA300_RS02145
	set-36									SAUSA300_RS02150
	set-37									SAUSA300_RS02155
	set-38									SAUSA300_RS02160

	set-39			CCIJOALM_00051						SAUSA300_RS02165
	set-40									SAUSA300_RS02185
**TSST1**	*tsst1*	SA_RS10445	SAHV_RS10855					SAV_RS10990		
Exoenzyme	**15 genes**	**14 genes**	**14 genes**	**14 genes**	**8 genes**	**9 genes**	**13 genes**	**14 genes**	**13 genes**	**15 genes**
**Hyaluronate lyase**	*hysA*	SA_RS11535	SAHV_RS11910	CCIJOALM_01836	orf01003; orf02344	MMEFEJFM_00763	JCKDIPHL_00142	SAV_RS12045	MW_RS11590	SAUSA300_RS11915
**Lipase**	*lip*	SA_RS14100	SAHV_RS14440	CCIJOALM_01431	orf00299	MMEFEJFM_02179	JCKDIPHL_00592	SAV_RS14580	MW_RS14055	SAUSA300_RS14475
	geh	SA_RS01780	SAHV_RS01635	CCIJOALM_00134	orf01336	MMEFEJFM_01640	JCKDIPHL_02456	SAV_RS01780	MW_RS01565	SAUSA300_RS01705
**Serine protease splABCDEF**	*splA*	SA_RS09180	SAHV_RS09600	CCIJOALM_02314			JCKDIPHL_02393	SAV_RS09735	MW_RS09370	SAUSA300_RS09620
	splB	SA_RS09175	SAHV_RS09595	CCIJOALM_02313			JCKDIPHL_02394	SAV_RS09730	MW_RS09365	SAUSA300_RS09615
	splC	SA_RS09170	SAHV_RS09590	CCIJOALM_02312			JCKDIPHL_02395	SAV_RS09725	MW_RS09360	SAUSA300_RS09610
	splD	SA_RS09165	SAHV_RS09585	CCIJOALM_02311			JCKDIPHL_02396	SAV_RS09720		SAUSA300_RS09605
	splE									SAUSA300_RS09600
	splF	SA_RS09160	SAHV_RS09580	CCIJOALM_02310				SAV_RS09715	MW_RS09355	SAUSA300_RS09595
**Staphopain**	*sspB*	SA_RS05105	SAHV_RS05515	CCIJOALM_00911	orf00437	MMEFEJFM_02043	JCKDIPHL_01339	SAV_RS05650	MW_RS05000	SAUSA300_RS05105
	sspC	SA_RS05100	SAHV_RS05510	CCIJOALM_00912	orf00436	MMEFEJFM_02042	JCKDIPHL_01338	SAV_RS05645	MW_RS04995	SAUSA300_RS05100
**Staphylocoagulase**	*coa*	SA_RS01295	SAHV_RS01150	CCIJOALM_00222	orf02188	MMEFEJFM_01303	JCKDIPHL_00044	SAV_RS01295	MW_RS01070	SAUSA300_RS01180
**Staphylokinase**	*sak*	SA_RS10100	SAHV_RS10515	CCIJOALM_00826		MMEFEJFM_01514	**JCKDIPHL_02208**	SAV_RS10650	MW_RS10280	SAUSA300_RS10540
**Thermonuclease nuc**	*nuc*	SA_RS06590	SAHV_RS04325	CCIJOALM_01953	orf00706	MMEFEJFM_00273; MMEFEJFM_01218	JCKDIPHL_01897	SAV_RS04460	MW_RS06500	SAUSA300_RS04185
**V8 protease**	*sspA*	SA_RS05110	SAHV_RS05520	CCIJOALM_00910	orf00438	MMEFEJFM_02044	JCKDIPHL_01340	SAV_RS05655	MW_RS05005	SAUSA300_RS05110
Immune modulation	**7 genes**	**7 genes**	**6 genes**	**7 genes**	**6 genes**	**7 genes**	**6 genes**	**6 genes**	**6 genes**	**7 genes**
**AdsA**	*adsA*	SA_RS00265	SAHV_RS00125	CCIJOALM_01365	orf00361	MMEFEJFM_02384	JCKDIPHL_00658	SAV_RS00270	MW_RS00125	SAUSA300_RS00130
**Capsule**		SA_RS00905,<br>SA_RS00900,<br>SA_RS00895,<br>SA_RS00890,<br>SA_RS00910,<br>SA_RS00915,<br>SA_RS00920,<br>SA_RS00925,<br>SA_RS00930,<br>SA_RS00935,<br>SA_RS00940,<br>SA_RS00945,<br>SA_RS00950,<br>SA_RS00955,<br>SA_RS00960,<br>SA_RS00965	SAHV_RS00745,<br>SAHV_RS00750,<br>SAHV_RS00755,<br>SAHV_RS00760,<br>SAHV_RS00765,<br>SAHV_RS00770,<br>SAHV_RS00775,<br>SAHV_RS00780,<br>SAHV_RS00785,<br>SAHV_RS00790,<br>SAHV_RS00795,<br>SAHV_RS00800,<br>SAHV_RS00805,<br>SAHV_RS00810,<br>SAHV_RS00815,<br>SAHV_RS00820	CCIJOALM_00472;<br>CCIJOALM_00473;<br>CCIJOALM_00474;<br>CCIJOALM_00475;<br>CCIJOALM_00476;<br>CCIJOALM_00477;<br>CCIJOALM_00478;<br>CCIJOALM_00479;<br>CCIJOALM_00480;<br>CCIJOALM_00481;<br>CCIJOALM_00482;<br>CCIJOALM_00483;<br>CCIJOALM_00484;	orf02115;<br>orf02116;<br>orf02117;<br>orf02118;<br>orf02119;<br>orf02120;<br>orf02121;<br>orf02122;<br>orf02123;<br>orf02124;<br>orf02125;<br>orf02127;<br>orf02128;<br>orf02129;<br>orf02130	MMEFEJFM_01363;<br>MMEFEJFM_01364;<br>MMEFEJFM_01365;<br>MMEFEJFM_01366;<br>MMEFEJFM_01367;<br>MMEFEJFM_01368;<br>MMEFEJFM_01369;<br>MMEFEJFM_01370;<br>MMEFEJFM_01371;<br>MMEFEJFM_01372;<br>MMEFEJFM_01373;<br>MMEFEJFM_01374;<br>MMEFEJFM_01375;	JCKDIPHL_02607;<br>JCKDIPHL_02608;<br>JCKDIPHL_02609;<br>JCKDIPHL_02610;<br>JCKDIPHL_02611;<br>JCKDIPHL_02612;<br>JCKDIPHL_02613;<br>JCKDIPHL_02614;<br>JCKDIPHL_02615;<br>JCKDIPHL_02616;<br>JCKDIPHL_02617;<br>JCKDIPHL_02618;<br>JCKDIPHL_02619;	SAV_RS00910,<br>SAV_RS00905,<br>SAV_RS00900,<br>SAV_RS00895,<br>SAV_RS00890,<br>SAV_RS00915,<br>SAV_RS00920,<br>SAV_RS00925,<br>SAV_RS00930,<br>SAV_RS00935,<br>SAV_RS00940,<br>SAV_RS00945,<br>SAV_RS00950,<br>SAV_RS00955,<br>SAV_RS00960,<br>SAV_RS00965	MW_RS00680,<br>MW_RS00675,<br>MW_RS00670,<br>MW_RS00665,<br>MW_RS00660*,<br>MW_RS00685,<br>MW_RS00690,<br>MW_RS00695,<br>MW_RS00700,<br>MW_RS00705,<br>MW_RS00710,<br>MW_RS00715,<br>MW_RS00720,<br>MW_RS00725,<br>MW_RS00730,<br>MW_RS00735	SAUSA300_RS00825,<br>SAUSA300_RS00815,<br>SAUSA300_RS00810,<br>SAUSA300_RS00805,<br>SAUSA300_RS00830,<br>SAUSA300_RS00835,<br>SAUSA300_RS00840,<br>SAUSA300_RS00845,<br>SAUSA300_RS00850,<br>SAUSA300_RS00855,<br>SAUSA300_RS00860,<br>SAUSA300_RS00865,<br>SAUSA300_RS00870,

				CCIJOALM_00485; CCIJOALM_00486; CCIJOALM_00487		MMEFEJFM_01376; MMEFEJFM_01377; MMEFEJFM_01378	JCKDIPHL_02620; JCKDIPHL_02621; JCKDIPHL_02622			SAUSA300_RS00875, SAUSA300_RS00880
**Cell wall associated fibronectin binding protein ebh**	*ebh*	SA_RS15265*	SAHV_RS15585*	CCIJOALM_01752	orf00812	MMEFEJFM_01089	JCKDIPHL_01114	SAV_RS15755*	MW_RS07115	SAUSA300_RS07235
**CHIPS**	*chp*	SA_RS10090		CCIJOALM_00828		MMEFEJFM_01512				SAUSA300_RS10530
**Fibrinogen binding protein efb**	*efb*	SA_RS05690	SAHV_RS06100	CCIJOALM_01635	orf00542	MMEFEJFM_01769	JCKDIPHL_01512	SAV_RS06235	MW_RS05585	SAUSA300_RS05690
**Sbi**	*sbi*	SA_RS12660	SAHV_RS13040	CCIJOALM_02065	orf00049	MMEFEJFM_00971	JCKDIPHL_00345	SAV_RS13175	MW_RS12680	SAUSA300_RS13060
**SCIN**	*scn*	SA_RS10085	SAHV_RS10505	CCIJOALM_00829	orf01257	MMEFEJFM_01511	JCKDIPHL_02209	SAV_RS10640	MW_RS10265	SAUSA300_RS10525
Biofilm	**5 genes**	**5 genes**	**5 genes**	**5 genes**	**4 genes**	**5 genes**	**5 genes**	**5 genes**	**5 genes**	**4 genes**
**Intercellular adhesion proteins**	*icaR*	SA_RS14075	SAHV_RS14415	CCIJOALM_01436	orf00295	MMEFEJFM_02184	JCKDIPHL_00587	SAV_RS14555	MW_RS14030	SAUSA300_RS14450
	icaA	SA_RS14080	SAHV_RS14420	CCIJOALM_01435	orf00296	MMEFEJFM_02183	JCKDIPHL_00588	SAV_RS14560	MW_RS14035	SAUSA300_RS14455
	icaD	SA_RS14085	SAHV_RS14425	CCIJOALM_01434		MMEFEJFM_02182	JCKDIPHL_00589	SAV_RS14565	MW_RS14040	
	icaB	SA_RS14090	SAHV_RS14430	CCIJOALM_01433	orf00297	MMEFEJFM_02181	JCKDIPHL_00590	SAV_RS14570	MW_RS14045	SAUSA300_RS14465
	icaC	SA_RS14095	SAHV_RS14435	CCIJOALM_01432	orf00298	MMEFEJFM_02180	JCKDIPHL_00591	SAV_RS14575	MW_RS14050	SAUSA300_RS14470
**Total genes**	**120 genes**	**77 genes**	**77 genes**	**72 genes**	**48 genes**	**56 genes**	**64 genes**	**75 genes**	**75 genes**	**71 genes**
