# Supplementary material for: Fitness Burden for the Stepwise Acquisition of First- and Second-Line Antimicrobial Reduced-Susceptibility in High-Risk ESKAPE MRSA Superbugs
Source: Antibiotics (Basel). 2025 Feb 28;14(3):244. doi: 10.3390/antibiotics14030244 (PMC11939686; doi:10.3390/antibiotics14030244)
Supplement: Supplementary file 1 [file antibiotics-14-00244-s001.zip › antibiotics-3486475-Table S4.pdf]

Table. S4: Generation times and Growth-rates of MRSA couples

ISOGENIC STRAIN-PAIRS	FITNESS COSTS			
	GROWTH-RATE			
	INDEPENDENT-GROWTH			
		Hourly generation Time (gT) (h:min)		Hourly Growth-Rates (r/h)
	T1	17.67	T1	2.13
	T2	28.5	T2	1.46
	T3	42.3	T3	0.98
**1-S Ref HA-MRSA DAP-S GSSA**	T4	21.44	T4	1.94
	T5	19.17	T5	2.17
	T6	18.54	T6	2.3
	T7	11.93	T7	3.48
	T24	-9h:40	T24	-0.07
	T1	41	T1	1.05
	T2	32.3	T2	1.288
	T3	42.3	T3	0.98
**1-R Ref HA-MRSA N315 DAP-S hGISA**	T4	50.2	T4	0.83
	T5	16.35	T5	2.51
	T6	48.5	T6	0.857
	T7	20.96	T7	1.9
	T24	11h:19	T24	0.06
	T1	37.4	T1	1.1
	T2	2h:31	T2	0.274
	T3	21.37	T3	1.9
**2-S HA-MRSA N315 DAP-S GSSA**	T4	37.86	T4	1.0
	T5	21.1	T5	1.97
	T6	35.1	T6	1.18
	T7	21	T7	1.98
	T24	15:11	T24	0.04
	T1	5h:11	T1	0.13
	T2	24.9	T2	1.67
	T3	1h:48	T3	0.386
**2-R HA-MRSA N315 DAP-R GSSA**	T4	37.86	T4	1
	T5	16.66	T5	2.49
	T6	38.24	T6	1.088
	T7	12.14	T7	3.426
	T24	12:29	T24	0.058
**3-S LA-MRSA ST398 DAP-S GSSA**	T1	1h:43	T1	0.40
	T2	23.2	T2	1.79
	T3	34.51	T3	1.2

	T4	23.98	T4	1.735
	T5	30.3	T5	1.3
	T6	23.5	T6	1.77
	T7	33.3	T7	1.29
	T24	5h:46	T24	0.12
**3-R LA-MRSA ST398 DAP-R hGISA**	T1	31.6	T1	1.56
	T2	1h:16	T2	0.6
	T3	50.7	T3	0.8
	T4	32.7	T4	1.27
	T5	32.9	T5	1.26
	T6	22.5	T6	1.85
	T7	31.46	T7	1.32
	T24	5h:43	T24	0.121
**4-S HA-MRSA DAP-S GSSA**	T1	1h:42	T1	0.41
	T2	34.54	T2	1.2
	T3	18.06	T3	2.30
	T4	26.4	T4	1.59
	T5	24.7	T5	1.7
	T6	13.8	T6	2.99
	T7	43.5	T7	0.95
	T24	-5h:32	T24	-0.12
**4-R HA-MRSA DAP-R hGISA**	T1	3h:48	T1	0.18
	T2	16.06	T2	2.59
	T3	36.5	T3	1.14
	T4	1h	T4	0.6
	T5	12	T5	3.5
	T6	19.42	T6	2.14
	T7	3h:14	T7	0.21
	T24	-17h	T24	-0.04
**5-S CA-MRSA DAP-S GSSA**	T1	23.98	T1	1.73
	T2	1h:25	T2	0.42
	T3	10.5	T3	3.96
	T4	12.5	T4	3.1
	T5	12.4	T5	3.2
	T6	12.1	T6	3.43
	T7	12.35	T7	3.3
	T24	2h:2	T24	0.37
**5-R CA-MRSA DAP-R GISA**	T1	48.7	T1	0.8
	T2	1h:45	T2	0.39
	T3	48:6	T3	0.856
	T4	20.64	T4	2.015
	T5	42,4	T5	0,98
	T6	15.36	T6	2.71

	T7	16.06	T7	2.59
	T24	2h:16	T24	0.35
**6-S Ref HA-MRSA DAP-S GSSA**	T1	17.67	T1	2.13
	T2	28.5	T2	1.46
	T3	42.3	T3	0.98
	T4	21.44	T4	1.94
	T5	19.17	T5	2.17
	T6	18.54	T6	2.3
	T7	11.93	T7	3.48
	T24	-9h:40	T24	-0.07
**6-R Ref HA-MRSA DAP-R GISA**	T1	30	T1	1.38
	T2	24.9	T2	1.66
	T3	12h:35	T3	0.05
	T4	18.66	T4	2.23
	T5	1h:36	T5	0.6
	T6	2h:38	T6	0.26
	T7	14.07	T7	2.9
	T24	9h:55	T24	0.069
