# Supplementary material for: Fitness Burden for the Stepwise Acquisition of First- and Second-Line Antimicrobial Reduced-Susceptibility in High-Risk ESKAPE MRSA Superbugs
Source: Antibiotics (Basel). 2025 Feb 28;14(3):244. doi: 10.3390/antibiotics14030244 (PMC11939686; doi:10.3390/antibiotics14030244)
Supplement: Supplementary file 1 [file antibiotics-14-00244-s001.zip › antibiotics-3486475-Table S5.pdf]

Table. S5: Competitive growth of MRSA couples

	1-S HA-MRSA DAP-S GSSA	1-R HA-MRSA DAP-S hGISA	2-S HA-MRSA DAP-S GSSA	2-R HA-MRSA DAP-R GSSA	3-S LA-MRSA DAP-S GSSA	3-R LA-MRSA DAP-R hGISA	4-S HA-MRSA DAP-S GSSA	4-R HA-MRSA DAP-R hGISA	5-S CA-MRSA DAP-S GSSA	5-R CA-MRSA DAP-R GISA	6-S HA-MRSA DAP-S GSSA	6-R HA-MRSA DAP-R GISA
**T0**	1,90E+05	5,80E+05	2,50E+05	2,80E+05	1,50E+05	1,50E+05	1,00E+05	1,00E+05	1,23E+05	2,28E+05	1,90E+05	2,50E+05
**T1**	2,74E+06	6,00E+04	6,75E+05	3,25E+05	8,40E+05	6,20E+04	1,40E+05	1,10E+05	1,26E+06	2,43E+05	3,36E+06	4,00E+04
**T2**	3,82E+06	8,00E+04	1,80E+06	2,00E+05	1,84E+06	1,60E+05	1,40E+06	1,00E+05	1,07E+07	1,30E+05	5,78E+06	1,60E+04
**T3**	4,60E+07	1,00E+06	2,30E+07	2,00E+06	1,47E+07	8,00E+05	6,60E+06	6,00E+05	2,54E+07	9,27E+05	4,10E+07	1,80E+04
**T4**	2,73E+08	7,20E+06	1,78E+07	3,20E+06	4,74E+07	6,20E+05	1,90E+08	3,60E+06	1,00E+08	2,70E+06	3,29E+08	8,00E+05
**T5**	4,79E+09	1,00E+07	1,23E+08	2,66E+07	1,16E+08	3,65E+06	1,70E+08	3,20E+07	2,50E+08	1,00E+07	6,00E+09	4,00E+06
**T6**	2,58E+09	2,30E+07	6,20E+08	1,40E+08	4,26E+08	3,40E+07	1,30E+09	4,10E+08	1,13E+09	1,12E+07	5,35E+09	5,00E+07
**T7**	3,66E+10	4,50E+08	5,00E+08	2,00E+09	1,70E+10	2,00E+09	2,70E+09	1,00E+08	1,29E+10	5,50E+07	1,20E+12	2,00E+08
**T24**	8,48E+09	2,00E+07	4,00E+08	1,70E+09	2,45E+09	1,50E+08	5,8E+08	2,0E+08	1,42E+12	5,10E+08	4,29E+10	1,20E+08
