# Supplementary material for: Fitness Burden for the Stepwise Acquisition of First- and Second-Line Antimicrobial Reduced-Susceptibility in High-Risk ESKAPE MRSA Superbugs
Source: Antibiotics (Basel). 2025 Feb 28;14(3):244. doi: 10.3390/antibiotics14030244 (PMC11939686; doi:10.3390/antibiotics14030244)
Supplement: Supplementary file 1 [file antibiotics-14-00244-s001.zip › antibiotics-3486475-Table S6,Figure.S1, S2 and S3.pdf]

Table. S6: *in-vitro* virulence factor analysis data

	1-S	1-R	2-S	2-R	3-S	3-R	4-S	4-R	5-S	5-R	6-S	6-R	USA 300	MW2
**Biofilm**	0.5	0.15	0.16	0.16	-	-	-	-	-	-	0.5	0.18	0.15	0.35
**α-hemolysis**	-	-	+	+	+	+	+	+	+	-	-	-	+	+
**β-hemolysis**	-	-	-	-	-	-	-	-	-	-	-	-	-	-
**δ-hemolysis**	-	-	-	-	-	-	-	-	+	-	-	-	+	+
**Staphyloxanthin**	++	+	+++	+	-	-	+	+	++	+	++	+	+	++
**Protease activity**	-	-	-	-	+	+	+	-	+	-	-	-	+	+
**Colony spreading diameter (mm)**	9	6	14	10	12	10	11	8	30	7	9	6	11	15

Legend: (- negative; + weak positive; ++ medium positive; +++ strong positive; ++++ high strong positive); Biofilm measurements at Optical Density 490 nm

Figure S1: α-hemolysin activity assay

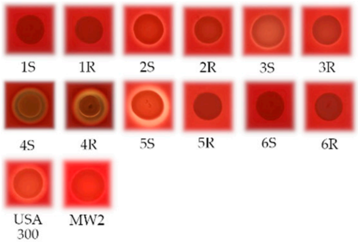

Legend: A positive activity was determined by the presence of a distinct clear halo surrounding the colonies

Figure S2: Staphyloxanthin production

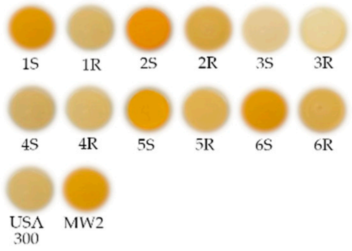

Legend: Positivity was considered with various degrees based on colony color ranging from yellow to orange

Figure S3: Caseinase production

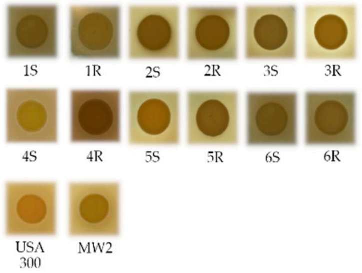

Legend: A clear halo around the colonies showed the degradation of caseins and the positivity of the assay
